# Editorial: Molecular Mechanisms of Flowering Plant Reproduction

**DOI:** 10.3389/fpls.2021.828136

**Published:** 2022-01-13

**Authors:** Natalia Pabón-Mora, Maria Helena S. Goldman, David R. Smyth, Jorge Muschietti, Maria Manuela R. Costa

**Affiliations:** ^1^Facultad de Ciencias Exactas y Naturales, Instituto de Biología, Universidad de Antioquia, Medellín, Colombia; ^2^Faculdade de Filosofia, Departamento de Biologia, Ciências e Letras de Ribeirão Preto, Universidade de São Paulo, São Paulo, Brazil; ^3^School of Biological Sciences, Monash University, Melbourne, VIC, Australia; ^4^Consejo Nacional de Investigaciones Científicas y Técnicas (CONICET), Buenos Aires, Argentina; ^5^Biosystems and Integrative Sciences Institute (BioISI), Plant Functional Biology Center, University of Minho, Braga, Portugal

**Keywords:** flower development, floral induction, flower meristem, organ identity, fertility, fruit set, seed viability

## Introduction

Plant reproduction is an intricate process important for the survival of all dominant autotrophs and critical in agriculture and stable food production as the basis of our diet. Angiosperms undergo extreme transformations during their ontogeny, leading to the reproductive transition. During this process, several steps are needed, including transforming a shoot apical or a lateral vegetative meristem into an inflorescence meristem with flowering competence. Floral meristems are formed from the latter accompanied by cell differentiation leading to gamete formation in specialized reproductive floral organs. Gamete interaction relies on successful pollination, whether that occurs *via* biotic or abiotic vectors. In addition, fertilization requires numerous molecular and hormonal signals in place and results in proper pollen tube growth, zygote viability, and seed formation. This Research Topic addresses some of the most outstanding discoveries on angiosperm reproduction in model species like *Arabidopsis thaliana* and *Oryza sativa* and crops like cauliflower, cassava, citrus, and sugarcane, among others. In the papers on the topic, the reader will discover highlights on extremely diverse floral promotion pathways, mechanisms of floral organ identity and morphogenesis, sporogenesis and gametogenesis, pollen presentation, pollination, and fertilization strategies.

## Flowering and The Fine-Tuning of Temperature and Light

The transition to reproduction starts by sensing the environmental signals, which act as checkpoints for molecular signals promoting the conversion to flowering. One of such key regulators is *FLOWERING LOCUS C* (*FLC*), a major floral repressor in *Arabidopsis thaliana*. Shirakawa et al. have identified a novel chemical compound, 4-Isoxazolecarboxylic acid, 3,5-dimethyl-2-(4-fluorophenyl)-4-isoxazole carboxylic acid 1-methyl-2-oxoethyl ester, named DEVERNALIZER01 (DVR01), that can decrease the deposited H3K27me3 in the *FLC* locus. Because these epigenetic marks are landmarks for epigenetic silencing of *FLC*, their discoveries will aid the study of chemically induced activation of *FLC* and the epigenetic control of this major locus during vernalization.

Flowering requirements have been more studied in model plants than in crops. In this Research Topic, Manechini et al. address the genetic mechanisms underlying photoperiodic induction in sugarcane. Their contribution compares differentially expressed genes in a cultivar subjected to florally inductive and non-inductive photoperiodic treatments. Their results provide a framework for comparing flowering pathways across grasses with unique requirements. They report upregulated processes and functions related to the response to abiotic stress, photoprotection, photosynthesis, light-harvesting, and pigment biosynthesis linked to inductive photoperiod. Similarly, the authors' report downregulated categories during flowering, including plant organ morphogenesis, shoot system development, macromolecule metabolic process, and lignin biosynthesis.

## The Floral Induction and the Formation of Floral Meristems: a Point of No Return During Reproductive Transitions

A key developmental landmark during reproductive transition is the formation of floral meristems. They specify a different fate, triggering morphological and anatomical transformations in each floral organ. Those shifts will lead to gamete and gametophyte development in specialized whorls. Cruz et al. report the expression of the *SCI1* (*Stigma/style cell-cycle inhibitor 1*) gene right after floral meristem (FM) specification of *Nicotiana tabacum*. *SCI1* is a cell proliferation regulator, co-expressed with major meristematic transcription factors like *WUSCHEL* (*WUS*) and *AGAMOUS* (*AG*). The authors show *NtSCI1* is expressed in all floral proliferative cells and is activated by NAG1, the AG of *N. tabacum*. They suggest SCI1 contributes to the regulation of floral meristematic activity termination.

Longevity is one key aspect of reproductive transitions. For instance, monocarpic plants having a single reproductive phase rely on a limited number of flower primordia during their lifespan to form a new generation. Wang et al. established morphological and genetic landmarks during bolting in *Arabidopsis thaliana* related to the control of stem cell population during the reproductive transition. Their study focuses on the spatiotemporal regulation of major controllers like *WUSCHEL* and *CLAVATA3*. In addition, this contribution highlights the role of reactive oxygen species (ROS) during this developmental transition.

## Organ Identity and Cell Fate Linked to Fertility and Gametogenesis

The study of male gametogenesis from stamen identity during floral organ development to specific cell fate acquisition before and after meiosis has been exciting. New data have emerged on the environmental conditions that can lead to male sterility. Similarly, the control of specific genetic mechanisms underlying female gamete formation has been recently addressed. This Research Topic comprises several contributions related to these themes in diverse taxa.

Audiences interested in the molecular control of accurate chromosome segregation during meiosis will find in the review by Saleme et al. an updated comprehensive summary on the role of the Anaphase-Promoting Complex/Cyclosome (APC/C) during reproduction. The APC/C is a multi-subunit complex that targets proteins for degradation *via* proteasome 26S. The functional characterization of APC/C subunits in *Arabidopsis* has revealed that all subunits investigated so far are essential for gametophytic development and/or embryogenesis.

Novel experimental data in *Arabidopsis* includes the exciting functional characterization of kinases and Armadillo homologs during gamete formation. Nibau et al. describe alternative splicing forms of the Cyclin-Dependent Kinase G1 (CDKG1) and show their involvement in pollen development and their role in maintaining fertility at high ambient temperature in *Arabidopsis*. Cabral et al. report that the Armadillo BTB Arabidopsis protein 1 (ABAP1) is not only important for cell division during vegetative growth, but it is critical during male and female gametophyte differentiation. The authors pinpoint and characterize ABAP1 complexes regulating microspore first asymmetric mitosis and polar nuclei fusion to form the central cell.

Fu et al. address the role of the rice homolog of the mammalian *BREAST CANCER 2* (*BRCA2*) gene. The authors show that *Osbrca2* mutant plants exhibit normal vegetative growth but experience complete male and female sterility due to severe meiotic defects. A careful dissection of the molecular pathways affected demonstrates that *OsBRCA2* is required for DNA double-strand break repair in meiotic cells, promoting accurate recombination.

Honsho et al. study the association between T2 RNases and the occurrence of gametophytic self-incompatibility (GSI) in citrus accessions. Their results carefully identify the homology between Japanese and Chinese citrus RNases and pinpoint clades involved in self-incompatibility.

Xu L. et al. provide a comprehensive atlas of *AUXIN RESPONSE FACTORS* (*ARFs*) expression during reproductive development in wheat (*Triticum aestivum L*.). They highlight the finding of three anther-specific *TaARF* genes (*TaARF8, TaARF9*, and *TaARF21*) whose expressions were significantly altered by low temperature in thermosensitive genic male-sterile (TGMS) wheat. The authors explain how these *TaARF* genes may participate in the cold-induced male sterility pathway and offer potential TGMS wheat breeding and hybrid seed production applications.

Sun et al. use a transcriptomic approach to identify key genes and regulatory networks affecting pollen maturation in rice anthers in response to different day lengths. Their research pinpoints to photoperiod- and temperature-sensitive genes affecting male fertility, *Carbon Starved Anthe*r and *UDP-glucose pyrophosphorylase*, respectively. Data presented by the authors provide a framework for identifying new environmentally sensitive genes regulating male fertility for use in crop improvement.

Xu X-F. et al. dive into the importance of accumulating magnesium (Mg) for proper pollen formation by studying magnesium transporter (MGT) members. The authors identify differences in growth rate between floral organs in *mgt6* mutant, including stamens, compared with wild-type (WT) and *MGT6*+*/*– plants. Their results indicate that slower bud development allows *mgt6* to accumulate sufficient amounts of Mg in the pollen, explaining why *mgt6* plants are fertile. Their research is critical for understanding how other redundant MGTs transport sufficient Mg for pollen formation.

Lee et al. provide an in-depth description of rice's AP180 N-terminal homology (ANTH) domain-containing proteins. The authors highlight that these proteins function as adaptive regulators for clathrin-mediated endocytosis in eukaryotic systems, and endocytosis is, at the same time, key during elongating pollen tubes. The authors emphasize that *OsANTH3*-mediated endocytosis is essential for rice pollen germination and *OsANTH3* mutants decreased seed fertility by reducing the pollen germination percentage in rice.

Developing up-to-date tools for hybrid breeding by cytoplasmic male sterile lines has been critical for remunerative agriculture in Indian cauliflowers (*Brassica oleracea* var. *botrytis* L.). The work by Singh et al. highlights the utility of organelle genome-based markers in distinguishing cytoplasm types in Indian cauliflowers and unveils the epistatic effects of the cytonuclear interactions influencing floral phenotypes.

In addition, pollen tube growth is essential for plant reproduction. This extended path involves female tissue recognition, rapid hydration and germination, polar growth, and tight cell wall synthesis and modification regulation. Its properties change not only along the pollen tube but also in response to guidance cues inside the pistil. In the review by Cascallares et al., readers can find the most recent advances in elucidating the molecular mechanisms involved in the regulation of cell wall synthesis and modification during pollen germination, pollen tube growth, and rupture.

## Carpel Development, Fruit Set, and Seed Viability

This Research Topic features many examples across flowering plants on the genetic mechanisms underlying the specialization of the female portion of the flower and its contribution to fertility. The review by Shen et al. comprehensively shows the advances in understanding carpel identity determination, morphogenesis, and floral meristem determinacy, as well as the role of transcription factors, hormones, and miRNAs during carpel formation in grasses. In addition, the authors discuss the conservation and divergence between the genetic and molecular aspects of carpel development between grasses and eudicots.

The research by Oluwasanya et al. focuses on the mechanistic factors regulating cassava flowering. The authors test the effect of combinations of plant growth regulators (PGRs) and pruning treatments for their effectiveness in improving flower production and fruit set in field conditions. The authors conclude that flower-enhancing treatments with pruning, the anti-ethylene PGR silver thiosulfate (STS), and cytokinin benzyladenine (BA) create widespread changes in the network of hormone signaling and regulatory factors beyond ethylene and cytokinin stimulating inflorescence and floral development.

Finally, the work by Vergès et al. takes us to the role of protein farnesylation in seed development. Farnesylation is a post-translational modification regulated by the *ERA1* (*Enhanced Response to ABA 1*) gene encoding the β-subunit of the protein farnesyltransferase in *Arabidopsis*. The authors pinpoint the contribution of farnesylation in multiple processes, including floral opening, pollen germination rates, storage protein, and fatty acid contents. Their work highlights the involvement of protein farnesylation in seed development and the control of traits of agronomic interest.

## Author Contributions

NP-M wrote the Editorial, while MG, DS, JM, and MC revised the text providing intellectual contributions. All authors contributed to the article and approved the submitted version.

## Conflict of Interest

The authors declare that the research was conducted in the absence of any commercial or financial relationships that could be construed as a potential conflict of interest.

## Publisher's Note

All claims expressed in this article are solely those of the authors and do not necessarily represent those of their affiliated organizations, or those of the publisher, the editors and the reviewers. Any product that may be evaluated in this article, or claim that may be made by its manufacturer, is not guaranteed or endorsed by the publisher.

